# Carotid Web Management in Symptomatic Patients: A Case Report and Literature Review

**DOI:** 10.7759/cureus.73857

**Published:** 2024-11-17

**Authors:** Hamza Asim, Tamer El-Nakhal, Mohammed Usman, Hannah Lines, Greg S McMahon

**Affiliations:** 1 Medical Education, University of Leicester, Leicester, GBR; 2 Vascular Surgery, University Hospitals Leicester, Leicester, GBR; 3 Internal Medicine, Warwick Hospital, Warwickshire, GBR; 4 Vascular Surgery, University Hospitals Coventry and Warwickshire, Coventry, GBR

**Keywords:** carotid duplex ultrasound, carotid web, ct angio, extracranial fibromuscular dysplasia, fibromuscular disease, internal carotid artery (ica), vascular anomaly, vascular surgery education

## Abstract

The carotid web is a rare fibromuscular dysplasia disease of the internal carotid artery wall. It is a cause of thromboembolic stroke in a demographic of patients generally younger than those with atherosclerotic carotid artery disease. It is easy to miss the diagnosis without a high index of suspicion. We present a case of a carotid web in a 36-year-old female who suffered a thromboembolic stroke as a result of an ipsilateral carotid web. This was managed with open surgical resection of the intimal web and bovine patch angioplasty. In addition to highlighting this pathology as a cause of internal carotid artery stenosis, we present a review of the literature and a consensus on management options.

## Introduction

Fibromuscular dysplasia of the carotid artery was first described in the literature in the 1960s, with Rainer et al. describing a young female with recurrent neurological symptoms caused by a discrete hyperplastic lesion in the left carotid artery [1].

The subsequent description of similar cases has led to the carotid web being defined as a part of fibromuscular dysplasia disease of the internal carotid artery wall. The exact aetiology remains theoretical, but it is regarded as a condition of a relatively younger stroke population [2].

The majority of ischaemic strokes are caused either by thromboembolism from a cardiac source/large artery atherosclerosis or by small vessel disease [3]. Management tends to focus on secondary prevention of stroke in patients with a known cause, in order to mitigate stroke recurrence, mortality and morbidity, as well as tackling short and long-term disability, especially in younger patients [4]. Carotid webs, as discussed herein, are one of the rarer causes of stroke and are presumed to be a source of carotid thromboembolism [3,4]. As well as highlighting this unusual pathology, we have reviewed the literature to update on the current consensus surrounding diagnosis and management [5].

## Case presentation

**Figure 1:**
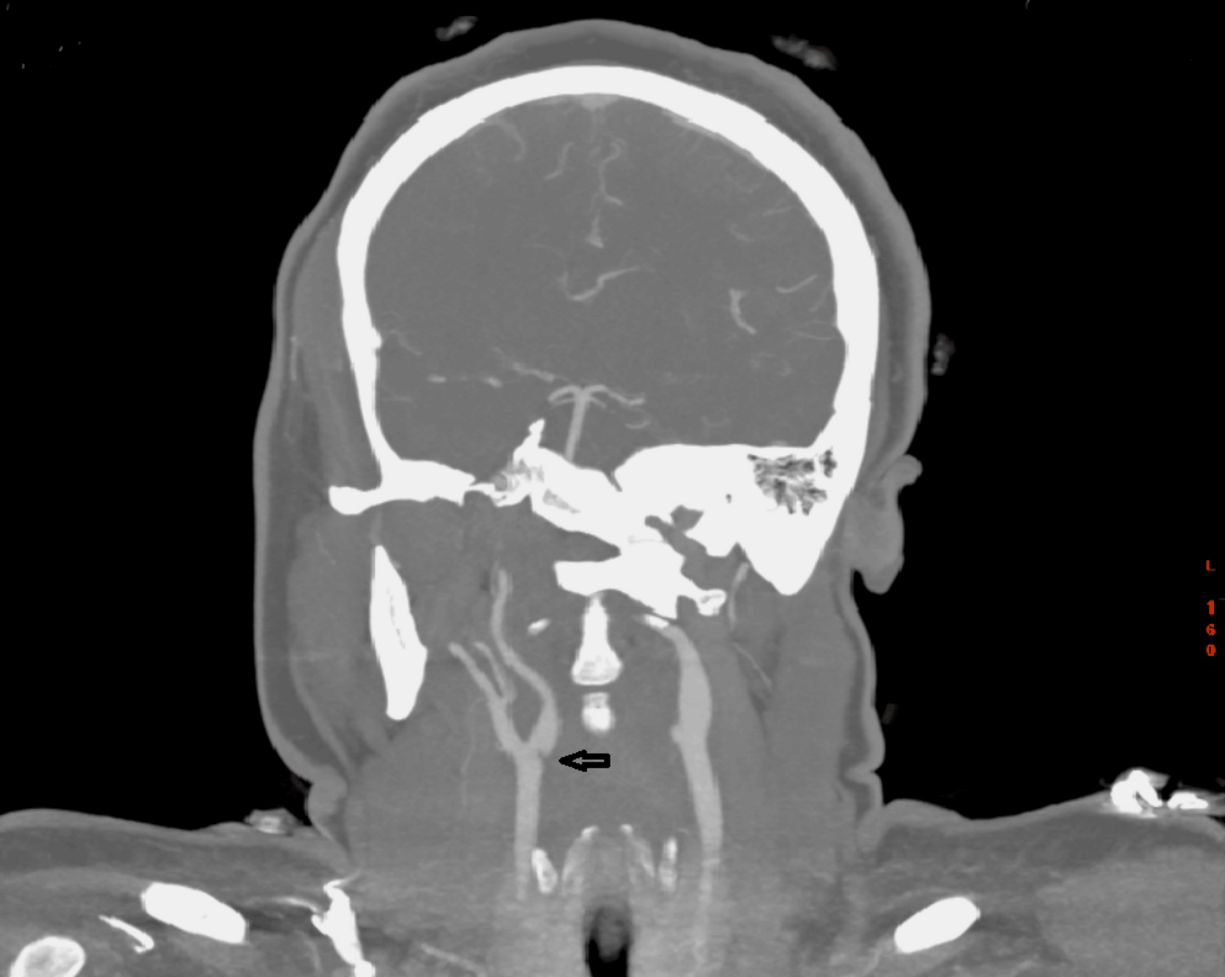
CT carotid angiogram showing a shelf-like structure in the right internal carotid artery (arrow)

**Figure 2:**
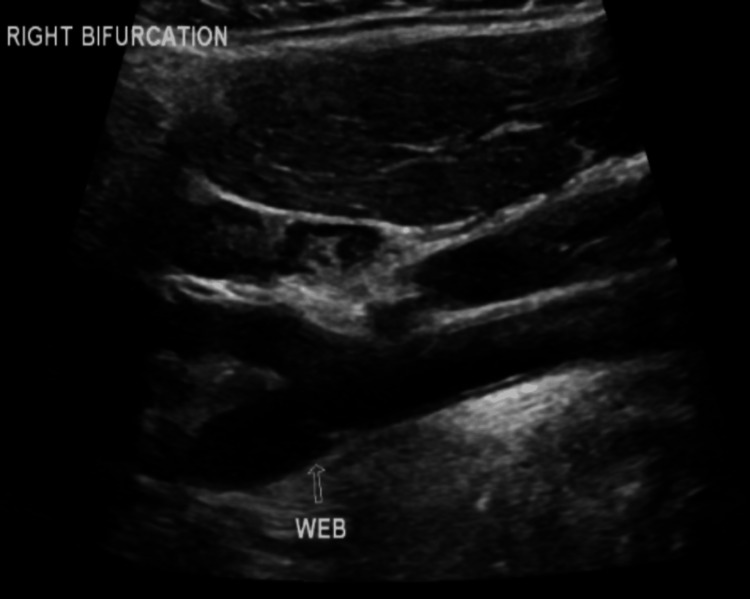
Proximal web only visible on B-mode of duplex ultrasound

**Figure 3:**
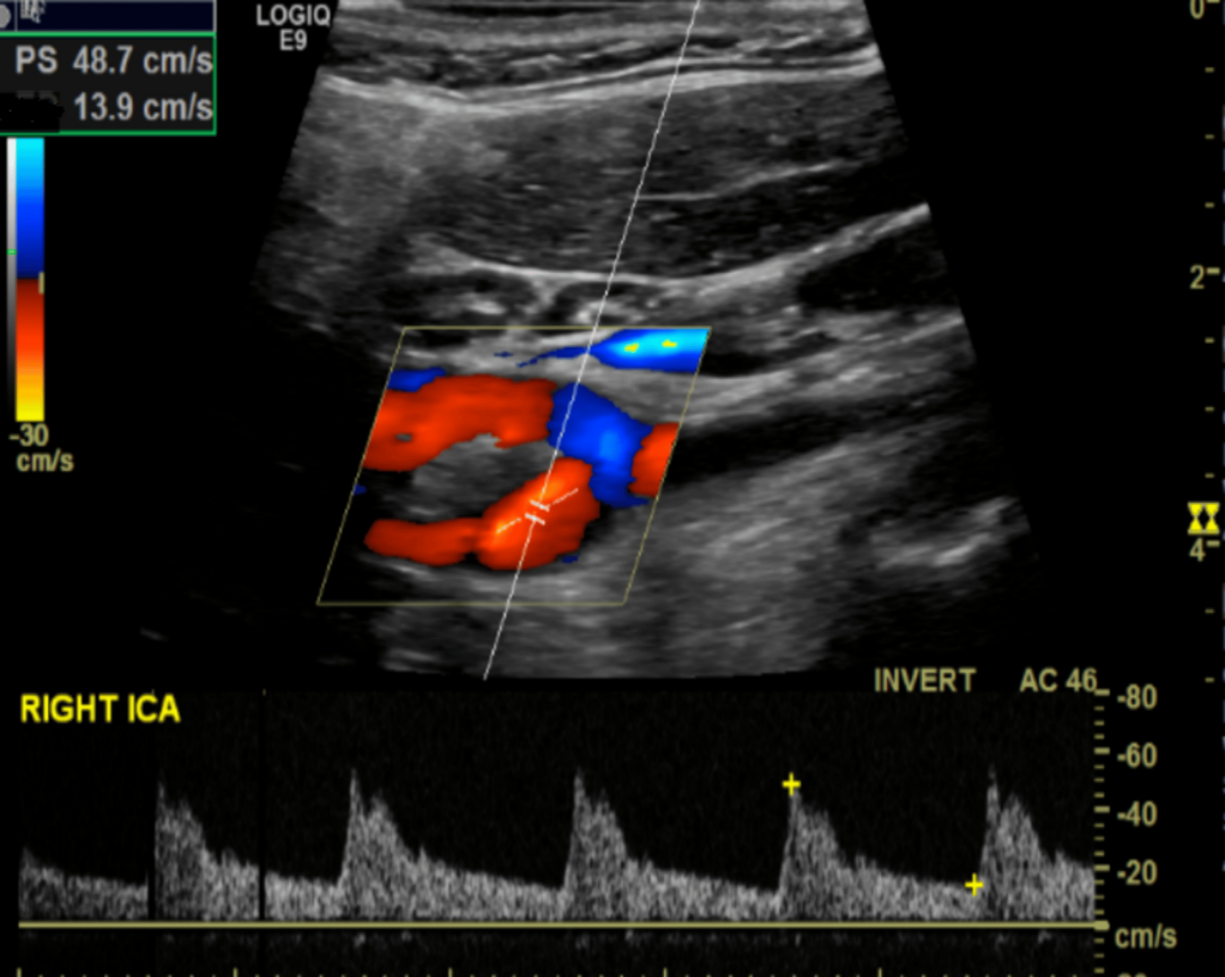
Small filling defect at bifurcation, normal flow and velocities; no evidence of turbulence

A 36-year-old female presented to the emergency department following a collapse at work. She had been incontinent of urine and on examination, had a dense left-sided weakness, left-sided inattention and a left homonymous hemianopia. A computer tomography (CT) scan of the head was performed, which revealed subtle loss of grey-white matter differentiation in the right insula, frontoparietal region and posterior temporal lobe in keeping with acute ischaemic change. On the same scan, note was made of a hyperdensity in the M1 segment of the right middle cerebral artery (MCA); on subsequent CT angiography (CTA), this had an appearance in keeping with intraluminal thrombus. This scan also revealed a smooth, transverse, “notch-like” filling defect in the posterior aspect of the right carotid bulb, just distal to the carotid bifurcation (Figure 1). There were no radiological features suggestive of carotid artery dissection and no mural calcification suggestive of atherosclerosis. Duplex ultrasound confirmed the filling defect in the right carotid bulb, showing a peak flow of 47.8 cm/s and no haemodynamic changes (Figures 2, 3). The carotid web was identified as the likely cause of the patient’s thromboembolic stroke. She underwent urgent thrombolysis and subsequently developed cerebral oedema leading to malignant MCA syndrome, necessitating urgent right decompressive craniectomy. The patient was discharged from the hospital to her own home after two months of inpatient care, with a Modified Rankin Score of 4. She required medical treatment for post-stroke seizures.

**Figure 4:**
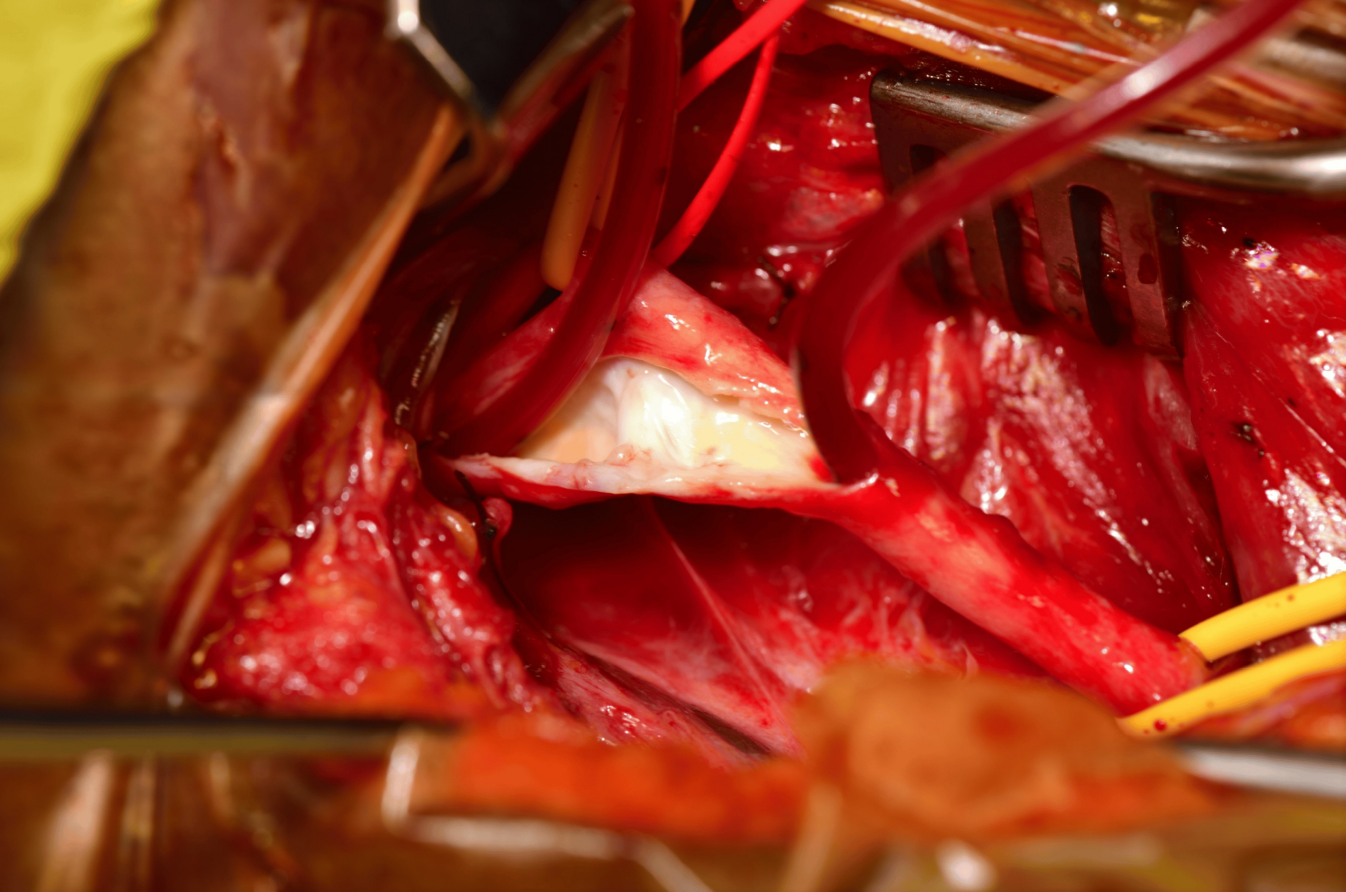
Intraoperative picture of right ICA carotid web with no other intimal atheroma or thrombus. ICA: internal carotid artery

At the initial vascular surgical review following the acute stroke, it was judged that the patient’s neurological recovery was insufficient to advocate prophylactic carotid surgery. However, by 21 months after the stroke, her Modified Rankin Score had decreased to 2 and it was therefore felt appropriate to offer her resection of the carotid web to reduce the risk of future stroke. She underwent open right carotid endarterectomy/carotid web resection and patch angioplasty (Figure 4), with intraoperative transcranial Doppler (TCD) assessment of the ipsilateral middle cerebral artery flows, the use of a Pruitt-Inahara shunt for the maintenance of ipsilateral cerebral flow while clamped and completion angioscopy.

The patient made an uncomplicated recovery from the operation. She was discharged within 48 hours with no immediate complications or other longer-term adverse events within the first 30 days and up to 6 months of follow-up.

## Discussion

The carotid web is a rare non-atherosclerotic and non-inflammatory flap-like fibromuscular dysplasia of the intima, causing a membrane that bulges intra-luminally within the carotid bulb. The immediate effect is internal carotid artery narrowing, impeding normal blood flow and increasing the risk of a thromboembolic event causing cerebral infarction. On radiological assessment, carotid webs are identified as an intra-luminal filling defect located along the posterolateral wall, which can mimic internal carotid artery stenosis [6].

The true incidence of the carotid web is unknown, but it is presumed to be a rare disease, accepting that underdiagnosis and misdiagnosis likely contribute to lower reporting. In an analysis of the 4137 patients in the Dutch MR CLEAN trial and registry participants, 30 were identified as likely to have a carotid web ipsilateral to the index ischaemic stroke. Their median age was 57 years, compared to the median age of 66 years for the patients with no carotid web as the cause for their large vessel occlusion. A higher proportion of the patients with ipsilateral carotid webs were female (73%) in comparison to the patients without a carotid web (40%), and 80% of the carotid webs in the sub-set were located in the right carotid artery [4].

Colour duplex ultrasonography is the current first-line imaging modality for the investigation of extracranial carotid artery disease [5], but it is operator-dependent, which could be a factor leading to carotid artery webs being misdiagnosed as isolated atherosclerotic plaques [7]. For example, in the presented case, the web was barely visible on B-mode ultrasound, and there was neither limitation to the internal carotid artery flow nor any haemodynamic turbulence. This finding could be due to the smoothness of the intimal surface into which the carotid web shapes itself. CTA is a more reliable modality that highlights the anatomical details in the carotid artery wall and can differentiate a carotid web from an atheroma, of course lacking the advantage of flow dynamics interpretation [7,8]. A recent study of the multimodality imaging of carotid webs compared CTA as the gold standard against both duplex ultrasonography and digital subtraction angiography (DSA) and showed inferior performance of ultrasound [9].

In a study of symptomatic patients with a carotid web managed conservatively, one in six experienced a recurrent stroke within 24 months [4]. Given this high rate, and in the absence of robust evidence to the contrary, it would seem rational to follow the guidelines for managing thromboembolic strokes arising from atherosclerotic lesions; dual antiplatelet therapy certainly and perhaps an argument for statins to reduce the risk of secondary events. However, these are typically younger patients without conventional stroke risk factors. It nonetheless seems reasonable to consider definitive intervention to further reduce the stroke risk, either with open surgery or endovascular stenting, just as for an atherosclerotic aetiology. Of paramount consideration is the patient’s recovery from any disability resulting from the initial neurological event, as corroborated by their modified Rankin score. Since carotid webs are more common in a population of relatively younger patients, this group might be expected to have a greater life-long risk of stroke recurrence, with preventative intervention, therefore, being more likely to be of benefit. But a recent study suggested that the surgical risk of open surgery in patients with a recent moderate-severe ischaemic stroke due to carotid artery stenosis >50% North American Symptomatic Carotid Endarterectomy Trial (NASCET) criteria and a cerebral ischaemic lesion of volume >4000 mm^3^ is high in the first two weeks. However, this reversed once a period of four weeks had elapsed, at which stage, the benefit of carotid endarterectomy was significant; the risk remains unknown in cases of carotid web ischaemic stroke patients [10].

While intervention for patients with recently symptomatic carotid webs would seem a reasonable approach, the management of asymptomatic patients presents more of a conundrum. The phenomenon is under-reported to the extent that no meaningful interpretation can be implied, although there are no reports of ischaemic neurological events after the incidental finding of a carotid web, notwithstanding the inevitability that each symptomatic carotid web was at one stage asymptomatic [2].

For the endovascular versus open management of carotid webs, there is insufficient evidence to promote one modality over the other. There are case reports of uncomplicated stenting, with follow-up to four years, and a systematic review suggested that neither stenting nor resection was associated with significant immediate or short-term complications, implying that both are safe and effective options [2]. This is evident in the 2023 ESVS guidelines, which suggested that for symptomatic patients with a carotid web in whom no other cause for stroke can be identified after detailed neurovascular workup, carotid endarterectomy or carotid artery stenting may be considered to prevent recurrent stroke [5]. The relative paucity of evidence coupled with the presumed requirement for long-term stent surveillance in a cohort of younger patients may, however, render the endovascular approach less favourable to the vascular surgeon than open resection [11].

## Conclusions

Carotid artery webs are a rare, but likely under-diagnosed cause of ischaemic strokes, for which there should be a high index of suspicion in younger patients without conventional stroke risk factors. Carotid artery webs are easily overlooked on ultrasound, and a second modality, such as CTA, is advisable before ruling out this aetiology. Recurrent stroke rates with symptomatic carotid artery webs are high, and either surgical or endovascular intervention should be considered to reduce the risk of future fatal or disabling stroke.
